# A case report of a patient with upper extremity symptoms: differentiating radicular and referred pain

**DOI:** 10.1186/1746-1340-15-10

**Published:** 2007-07-19

**Authors:** Clifford W Daub

**Affiliations:** 1 1120 Stelton Road, Piscataway, NJ 08854, USA

## Abstract

**Background:**

Similar upper extremity symptoms can present with varied physiologic etiologies. However, due to the multifaceted nature of musculoskeletal conditions, a definitive diagnosis using physical examination and advanced testing is not always possible. This report discusses the diagnosis and case management of a patient with two episodes of similar upper extremity symptoms of different etiologies.

**Case Presentation:**

On two separate occasions a forty-four year old female patient presented to a chiropractic office with a chief complaint of insidious right-sided upper extremity symptoms. During each episode she reported similar pain and parasthesias from her neck and shoulder to her lateral forearm and hand.

During the first episode the patient was diagnosed with a cervical radiculopathy. Conservative treatment, including manual cervical traction, spinal manipulation and neuromobilization, was initiated and resolved the symptoms.

Approximately eighteen months later the patient again experienced a severe acute flare-up of the upper extremity symptoms. Although the subjective complaint was similar, it was determined that the pain generator of this episode was an active trigger point of the infraspinatus muscle. A diagnosis of myofascial referred pain was made and a protocol of manual trigger point therapy and functional postural rehabilitative exercises improved the condition.

**Conclusion:**

In this case a thorough physical evaluation was able to differentiate between radicular and referred pain. By accurately identifying the pain generating structures, the appropriate rehabilitative protocol was prescribed and led to a successful outcome for each condition. Conservative manual therapy and rehabilitative exercises may be an effective treatment for certain cases of cervical radiculopathy and myofascial referred pain.

## Background

Among the general population, musculoskeletal pain and injury rank second only to upper respiratory conditions as the most common reasons for visiting a physician[1]. About 23% of patient visits to the family physician and 20% of visits to the emergency department are for musculoskeletal pain and injury [2-4].

Kahl reported that osteoarthritis is the single most common musculoskeletal problem, followed by isolated regional joint pain of the back, shoulder, knee and neck[4]. However, the frequency of patients presenting to physicians with many other specific conditions is not known.

Just as lower extremity pain, such as sciatica, often originates from the lumbo-pelvic region, upper extremity pain referred from the cervico-thoracic region is also common. For cervical radiculopathy, the rate has variably been shown to be 83.2 per 100,000 by Rabakrishnan et al. and 3.3 cases per 1,000 people by Wainner et al. [5,6]. However, there are many other musculoskeletal causes of upper extremity pain originating from the cervical-thoracic region such as peripheral neuropathy, vascular impingement (thoracic outlet syndrome) or myofascial syndromes and less is known about the epidemiology of these conditions.

The role of the physician is to identify as accurately as possible the pain generating tissues and determine appropriate treatment. Unfortunately, specific diagnosis of the cause of musculoskeletal pain is not always possible because we do not have valid and reliable physical examination tests for many conditions [7-10].

In addition, advanced testing such as diagnostic imaging is not diagnostic alone, but must be correlated with clinical exam and patient history due to poor specificity and the prevalence of clinically false-positive interpretations [11-13]. Even electrodiagnostic testing, which has high specificity for neuropathies, has been shown to be poorly sensitive to neuropathic pain in which there is not yet axonal damage and impaired conduction[14].

It has been argued that despite the lack of definitive diagnosis, once red flag signs for conditions such as tumor, infection and fracture have been ruled-out, a course of conservative treatment focused on restoring overall function is indicated[15].

The purpose of this case report is to discuss the differential diagnosis of a patient with two episodes of upper extremity pain and subsequently the conservative rehabilitative protocol used in each case.

## Case presentation

The patient is a forty four year old female who presented to a chiropractic office for evaluation and treatment of right upper extremity pain. She described the pain as starting in her neck and shoulder on the right and radiating down her right arm to her fingers. She also complained of tingling and numbness of her right lateral forearm and hand as well as loss of grip strength. She stated that the symptoms were insidious in onset several weeks prior with no history of trauma. The symptoms were constant and severe and getting worse in recent days. Working at her computer or using her right arm aggravated the condition, but she achieved some temporary relief with rest. She denied any prior upper extremity symptoms, but reported that she had experienced chronic frequent neck and upper trapezius pain on the right for years that was mild in nature and did not limit her activities of daily living or her job performance as a management information systems manager.

Physical examination demonstrated normal cervical ranges of motion. Upper extremity symptoms were increased with upper limb tension tests[6]. Valsalva test and neutral cervical compression were negative, but Spurling's test was positive on the right. Arm abduction provided relief of upper extremity symptoms. Manual muscle testing and deep tendon reflexes of the upper and lower extremities were normal bilaterally. Grip strength dynamometry revealed the following: 40/38/40 left and 60/58/60 right. The patient is right hand dominant. Palpation of the wrist extensors caused increased numbness of the first three digits of the right hand. Spinal palpation revealed segmental joint dysfunction at multiple levels in the cervical and thoracic spine with grade II tenderness at C4–5–6 on the right (tenderness ratings per American College of Rheumatology Pain Scale). Hypertonicity and grade I tenderness of the levator scapulae, anterior scalene and subocciptal musculature was noted on the right.

A radiographic examination of the cervical spine was also performed. The films were read by a radiologist and revealed a block vertebra at C2–3, a markedly reduced cervical lordosis, advanced discogenic spondylosis at C5–6 and moderate to advanced uncovertebral arthrosis at C5–6 which he noted could be associated with foraminal encroachment and C6 radiculopathy.

Based on the patient's history and the results of the physical and radiographic examinations a working diagnosis of cervical radiculopathy was formed. Differential diagnoses also included thoracic outlet syndrome and brachial neuritis associated with postural faults and segmental joint dysfunction.

Treatment included spinal manipulation to the restricted segments, post-isometric relaxation to the hypertonic musculature and manual long axis traction of the cervical spine above the level of the suspected nerve root involvement. As the radiculitis lessened and the severity of the patient's symptoms decreased she was also instructed on neuromobilization techniques to decrease possible nerve root adhesions[16,17].

The patient was treated eighteen times over a seven week period during which time she experienced progressive relief. At the end of that time the patient's upper extremity symptoms had resolved but she continued to experience mild neck and upper back pain and stiffness that she described as tolerable. She expressed satisfaction with her outcome and was released from rehabilitative care. During the next year she was seen periodically on a supportive basis for mild flare-ups of neck pain and stiffness and upper extremity parasthesias that were quickly resolved using the same therapies.

Approximately eighteen months after her initial symptoms the patient again experienced similar severe right upper extremity symptoms. Subsequently, the patient's primary medical physician referred her for an MRI of the cervical spine. The radiology report noted a disc osteophyte complex at C5–6 encroaching upon the subarachnoid space and right neural foramina. Consequently the patient was referred by her medical physician for a surgical consultation. However, the patient was resistant and wished to pursue conservative treatment and again presented to the chiropractic office.

At that time the patient had not been treated in the chiropractic office for almost five months. She complained of upper back and shoulder pain on the right as well as pain and numbness of the lateral forearm and hand that was persisting for several months. However, she noted that the intensity of the symptoms was not quite as severe as when she initially experienced the condition two years earlier. In intervening months she experienced occasional numbness of the distal right upper extremity, but did not report any pain.

Physical examination demonstrated normal cervical ranges of motion. Neutral cervical compression and Spurling's test were negative. Cervical distraction provided modest relief of the cervical spine symptoms, but had no effect on the upper extremity symptoms. The upper limb tension test produced anterior forearm pain, but did not reproduce the current chief complaint. However, digital palpation of a trigger point in the right infraspinatus muscle did exacerbate the chief complaint of shoulder pain and parasthesias of the lateral forearm and hand.

Based on the physical examination the cause of the patient's current complaint appeared to be myofascial referred pain from an active trigger point. Treatment focused on manual trigger point therapy, including both ischemic compression and post-isometric relaxation, as well as functional postural correction. Due to the chronic nature of the condition active rehabilitation included cervical retraction and mid/lower trapezius strengthening exercises. Cervical and thoracic spinal manipulation was also used to address segmental joint dysfunction. She was treated three times during a two week period and her upper extremity symptoms resolved. During the next six months the patient did not experience any upper extremity pain or parasthesia, though she reported intermittent cervico-thoracic pain and stiffness associated with sitting at her computer and work-related stress.

## Discussion

The initial purpose of the consultation and physical examination of the patient with a musculoskeletal complaint is to determine the pain generating structures. Historically there are many physical examination tests and procedures that have been developed and passed down from one clinician to another in the academic and clinical settings without systematic evaluation of validity[7,10].

While advanced testing such as MRI and electrodiagnostics have not been shown to be valid stand-alone diagnostic procedures, they can contribute to diagnostic accuracy [11-13]. However, due to the high cost and sometimes invasive nature of these tests there is great benefit in having the ability to accurately diagnose musculoskeletal conditions via low cost and time efficient consultation and physical exam.

As such, diagnostic criteria are being developed for certain conditions. Cervical radiculopathy has been defined as an impingement or inflammatory irritation of the cervical spine nerve root most commonly caused by cervical spondylosis or intervertebral disc herniation resulting in pain radiating along neural pathways of the upper extremity[5]. Historically, nerve root compression was indicated by abnormal muscle strength, deep tendon reflexes or dermatomal sensation. However, many patients are neurologically intact yet present with cervical radiculopathy symptoms due to inflammatory irritation of the nerve root. For these patients a different set of sensitive tests is required.

Recently Wainner et al. defined a group of clinical exam tests that could identify with 90% probability the likelihood of the presence of cervical radiculopathy[6]. The tests shown to be most useful for indicating cervical radiculopathy were the upper limb tension test, ipsilateral cervical rotation less than 60 degrees, neck distraction test and Spurling test[6]. Rubinstein et al, also recently completed a systematic review of the diagnostic accuracy of physical exam tests for cervical radiculopathy. They concluded that Spurling, neck distraction, Valsalva and upper limb tension tests are most useful in establishing a diagnosis of cervical radiculopathy in patients without neurological deficits[10].

**Figure 1:**
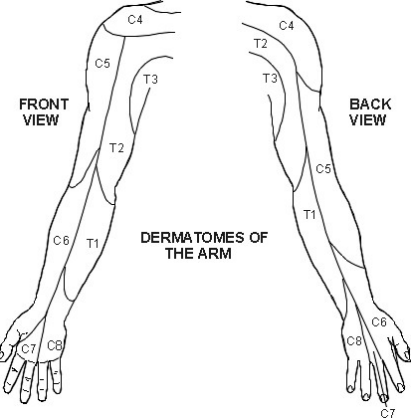
**Upper Extremity Dermatomes**. (Permission granted by PILs licensing to use figure – Diagram source copyright EMIS and PiP 2006)

The patient in this case report had positive Spurling, neck distraction and upper limb tension tests. In addition, arm abduction decreased the symptoms and palpation of C4–6 on the right reproduced the chief complaint along the lateral arm and forearm. [See Figure 1] The combination of these findings contributed to the chiropractor's confidence in a diagnosis of cervical radiculopathy and the decision to proceed with conservative therapy.

Subsequent treatment was designed to locally decrease the irritation of the involved nerve root and globally improve postural and segmental spinal biomechanics. A passive treatment protocol involving manipulation of the cervical and thoracic spine and manual cervical distraction, which has previously been shown to be effective for cervical radiculopathy, was initiated[16,18].

Within several treatments the patient began to experience a decrease in the intensity of the upper extremity symptoms. She was then also instructed on an active cervicobrachial neuromobilization technique which has been suggested can break perineural adhesions resulting from an inflammatory response in conditions such as cervical radiculopathy, thus aiding the healing process [16,17].

When the patient presented the second time to the chiropractor complaining of right upper extremity symptoms she also had the results of a cervical MRI completed three months prior demonstrating foraminal encroachment at C5–6 on the right.

**Figure 2:**
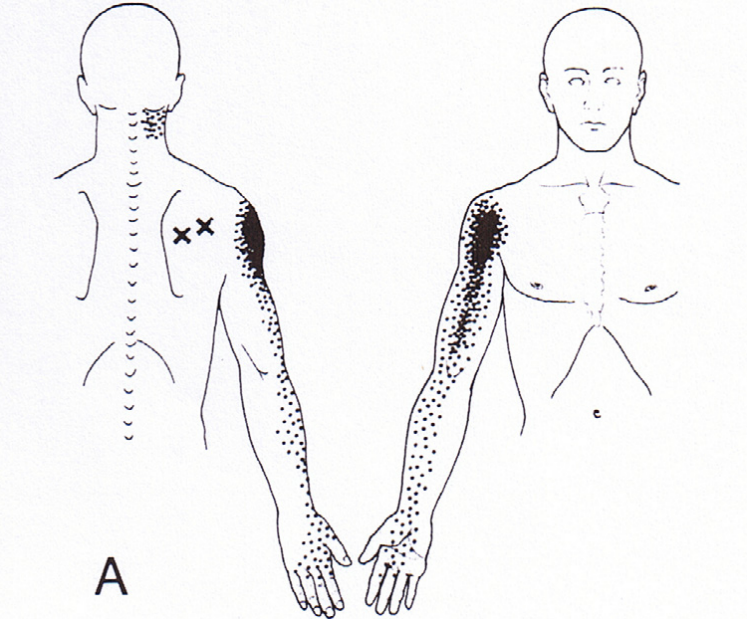
**Infraspinatus Trigger Point – Referred pain pattern**. (Permission granted by Lippincott Williams & Wilkins to use figure – Travell JG Simons DG. Myofascial Pain and Dysfunction 1983)

However, this time the chiropractor was unable to reproduce the chief complaint with the same physical exam tests as previously performed. Each of the cervical radiculopathy tests; Spurling's cervical compression, cervical distraction, arm abduction and upper limb tension, was negative. The patient was also neurologically intact with regard to muscle strength and deep tendon reflexes. The chief complaint was only reproduced by palpation of a trigger point in the right infraspinatus muscle [See Figure 2].

Myofascial trigger points have been defined as hyperirritable loci within taut bands of skeletal muscle that can produce local and referred pain[19]. Sciotti el al, have demonstrated that trigger points of the upper trapezius muscle can be reliably localized by a clinician using manual palpation[20].

While the MRI revealed anatomical changes consistent with potential causes of cervical radiculopathy, given the lack of clinical findings suggesting such, it is unlikely that the nerve root was compressed, irritated or inflamed during the second episode and therefore not the cause of symptoms. Because the pain patterns of a C6 cervical radiculopathy and infraspinatus trigger point are similar (See Figures 1 and 2), confusion can result if the clinician bases the diagnosis solely on imaging results and symptomology. Both must be correlated with the physical exam findings.

The differential diagnosis of radicular and referred myofascial pain is also complicated by the variable nature of pain patterns. Travell stated that pain referred from myofascial trigger points does not follow a simple pattern and may not always occur within the same dermatome, myotome or sclerotome[19]. Also, Bove et al. recently reported that radicular pain symptoms are perceived in deep structures rather than on the skin and that myotomal or sclerotomal patterns may be more diagnostic than traditional dermatomal charts[21].

In this case it is possible that the patient was presenting at different stages of functional pathology along a cervical radiculopathy continuum. The first episode may have represented a true nerve root irritation that was confirmed with provocative testing of the cervical spine. However, the second episode may have represented an earlier stage of cervical radiculopathy that while still causing neuropathic symptoms, may not be detected on physical examination if the irritation of the nerve root has not reached a certain threshold. It is unknown if the myofascial trigger point in the infraspinatus muscle in the second episode was a result of postural and biomechanical faults of the scapulothoracic region or if, given the infraspinatus muscle is innervated by the suprascapular nerve with contribution from the C5 and C6 nerve roots, that the muscle becomes hyperirritable due to nerve root compromise at these levels.

During the patient's second episode, she was treated with manual digital pressure to the trigger point as well as cervical distraction and spinal manipulation, so there was some duplication of treatment with the earlier acute radiculopathy. However, given the rapid response to treatment during the second episode compared with the first, it appears that the trigger point was the primary source of symptoms.

## Conclusion

Although diagnosis of musculoskeletal conditions is often not an exact science, in this case the physician was able to reproduce the chief complaint and use a test item cluster to identify the pain generating structures with good probability. This led to a conservative functional approach to rehabilitation that was successful in resolving episodes of both cervical radiculopathy and myofascial referred pain in one patient.

## Competing interests

The author(s) declare that they have no competing interests.
